# Ramipril Reduces Acylcarnitines and Distinctly Increases Angiotensin-Converting Enzyme 2 Expression in Lungs of Rats

**DOI:** 10.3390/metabo12040293

**Published:** 2022-03-26

**Authors:** Joanna Kosacka, Claudia Berger, Uta Ceglarek, Anne Hoffmann, Matthias Blüher, Nora Klöting

**Affiliations:** 1 Medical Department III—Endocrinology, Nephrology, Rheumatology, University of Leipzig Medical Center, Liebigstr. 20, 04103 Leipzig, Germany; claudi.berger@gmx.de (C.B.); bluma@medizin.uni-leipzig.de (M.B.); 2 Department of Visceral, Transplant, Thoracic and Vascular Surgery, University of Leipzig Medical Center, Liebigstr. 21, 04103 Leipzig, Germany; 3 Institute of Laboratory Medicine, Clinical Chemistry and Molecular Diagnostics, Leipzig University Medical Center, 04103 Leipzig, Germany; uta.ceglarek@medizin.uni-leipzig.de; 4 Helmholtz Institute for Metabolic, Obesity and Vascular Research (HI-MAG) of the Helmholtz Zentrum München at the University of Leipzig, Ph.-Rosenthal-Str. 27, 04103 Leipzig, Germany; anne.hoffmann@helmholtz-muenchen.de (A.H.); nora.kloeting@helmholtz-muenchen.de (N.K.)

**Keywords:** ACE2, lungs, Ramipril, SARS-CoV-2, C3M, C4M, C5M

## Abstract

The angiotensin-converting enzyme 2 (ACE2) receptor has been identified as the entry receptor for the severe acute respiratory syndrome coronavirus 2 (SARS-CoV-2) that is abundantly expressed in many organs. With respect to the role of circulating ACE2 and its receptor expression in the pathogenesis of the SARS-CoV-2 infection, it is still debated whether diseases such as hypertension or pharmacotherapies, including ACE inhibitors and angiotensin receptor blockers that affect ACE2 receptor expression, may modulate the severity and outcome of the coronavirus disease 2019 (COVID-19). We therefore tested the hypothesis that treatment with the ACE inhibitor Ramipril affects organ-specific ACE2 receptor mRNA and protein expression as well as the serum metabolome in BioBreeding (BB) rats. Twelve male BioBreeding rats were randomly divided into a Ramipril (10 mg/kg body weight) treatment group or a control group (*N* = 12; *n* = 6 per group) over a period of seven days. Ramipril treatment resulted in the reduction of acylcarnitines (C3–C6) out of 64 metabolites. Among the different organs studied, only in the lungs did Ramipril treatment significantly increase both *Ace2* mRNA and ACE2 receptor membrane protein levels. Increased ACE2 receptor lung expression after Ramipril treatment was not associated with differences in ACE2 serum concentrations between experimental groups. Our data provide experimental in vivo evidence that the ACE inhibitor Ramipril selectively increases pulmonary ACE2 receptor mRNA and protein levels and reduces acylcarnitines.

## 1. Introduction

Ramipril is an ACE-i (angiotensin-converting enzyme inhibitor) that is used for several indications such as hypertension or the prevention of heart failure progression [1]. In addition to diseases such as hypertension, treatment with inhibitors of the renin-angiotensin-aldosterone system (RAAS) causes an upregulation of ACE2 expression; this has led to the hypothesis that ACE inhibitor therapy may predispose patients to more severe COVID-19 courses [2,3,4,5,6]. Furthermore, the effects of Ramipril on the serum metabolome have not been systematically studied. The angiotensin-converting enzyme 2 (ACE2) receptor has been identified as an entry receptor for the severe acute respiratory syndrome coronavirus 2 (SARS-CoV-2) [7,8]. The ACE2 receptor is expressed on the membrane surface of several pulmonary and extra-pulmonary cell types, including cardiac, renal, intestinal, and endothelial cells [9]. On the other hand, circulating ACE2 exhibits protective effects on lung function in acute respiratory distress syndrome, which could be associated with less severe COVID-19 outcomes. In diabetes, circulating ACE2 seems to have a protective role in the progression of cardiovascular and renal complications and has been suggested as a potential therapeutic target for the management of diabetes and its complications [10,11]. Importantly, a recent meta-analysis including seven trials with 73,122 patients did not find significant associations between the intake of RAAS inhibitors and the likelihood of a positive COVID-19 test result, mortality, or severe illness from COVID-19 [12]. However, the effect of RAAS-inhibitor treatment on ACE2 receptors in the lungs in vivo is not clear [2,4,6,13]. Despite recent advances in our understanding of COVID-19 pathomechanisms, human experimental investigations can only give limited insights into the specific regulation of ACE2 expression. Therefore, we tested the hypothesis that seven days of treatment with the ACE inhibitor Ramipril alters serum metabolome parameters and the expression of *Ace2* receptors in an organ-specific manner in 12-week old BioBreeding/Ottawa Karlsburg Leipzig (BB/OKL) rats.

## 2. Results

**Figure 1:**
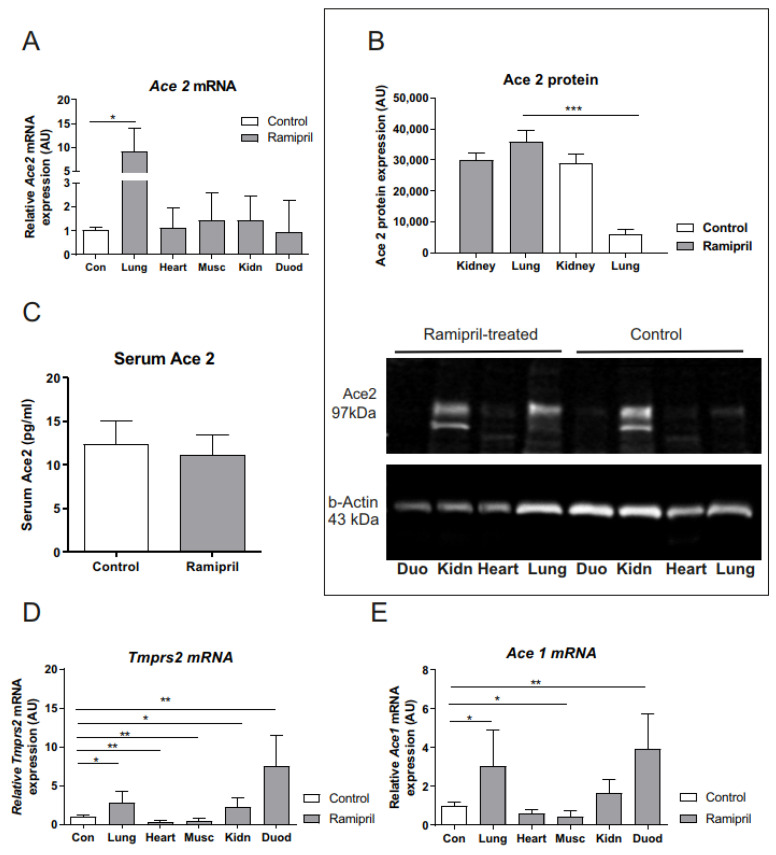
*Ace2*, *Tmprss2,* and *Ace1* tissue distributions. (**A**) *Ace2* mRNA receptor (*n* = 6 per experimental group); (**B**) membrane protein ACE2 receptor expression as well as below representative Western blot images in different organs of control (Con, *n* = 5) and Ramipril-treated (10 mg/kg BW/day; *n* = 5) BioBreeding/OKL rats. Expression of ACE2 receptor membrane protein was only detectable in kidneys and lungs. Therefore, only results for ACE2 receptors in kidneys and lungs are displayed; (**C**) Serum circulating ACE2 concentrations; (**D**) *Tmprss2* and (**E**) *Ace1* mRNA expression in different tissues of control (*n* = 6) and Ramipril-treated rats. Results are expressed as means ± SEM. The different degrees of significance were indicated as follows:* *p* < 0.05, ** *p* < 0.01, *** *p* < 0.001. Differences among the groups (data are normally distributed) were performed using one-way-ANOVA and the Newman–Keuls test (**A**,**B**,**D**,**E**) or *t*-test (**C**) with GraphPad Prism 9 Software (Jandel Scientific, San Rafael, CA, USA). Abbreviations: Con, -Control; Duo, -duodenum; Kidn, kidney; Musc, muscle; AU-arbitrary units.

Seven days of Ramipril treatment using a dosage that reflects the typical human dose for anti-hypertensive therapy did not cause changes in body weight (Table 1). After short-term Ramipril treatment, we find a significant, lung-specific increase in ACE2 receptors in both *Ace2* mRNA expression and ACE2 membrane protein levels compared to the control rats (Figure 1A,B). The ACE2 membrane protein was only expressed/detectable in lung and kidney tissue (30 µg per lane; Figure 1B). In contrast, ACE2 mRNA and protein levels in the heart, kidneys, duodenum, and muscle were not altered by Ramipril administration (Figure 1A,B). The elevated ACE2 protein expression in the lungs of rats treated with Ramipril was not associated with a higher concentration of circulating ACE2 in serum compared to controls (Figure 1C).

Androgen-regulated transmembrane protease serine 2 (*Tmprss2)* and *Ace1* mRNA were increased in the lungs, kidneys, and duodenums of rats treated with Ramipril compared to the controls (Figure 1D–E). As expected, *Ace1* mRNA was significantly reduced after Ramipril treatment in the heart and in muscle (Figure 1E).

**Figure 2:**
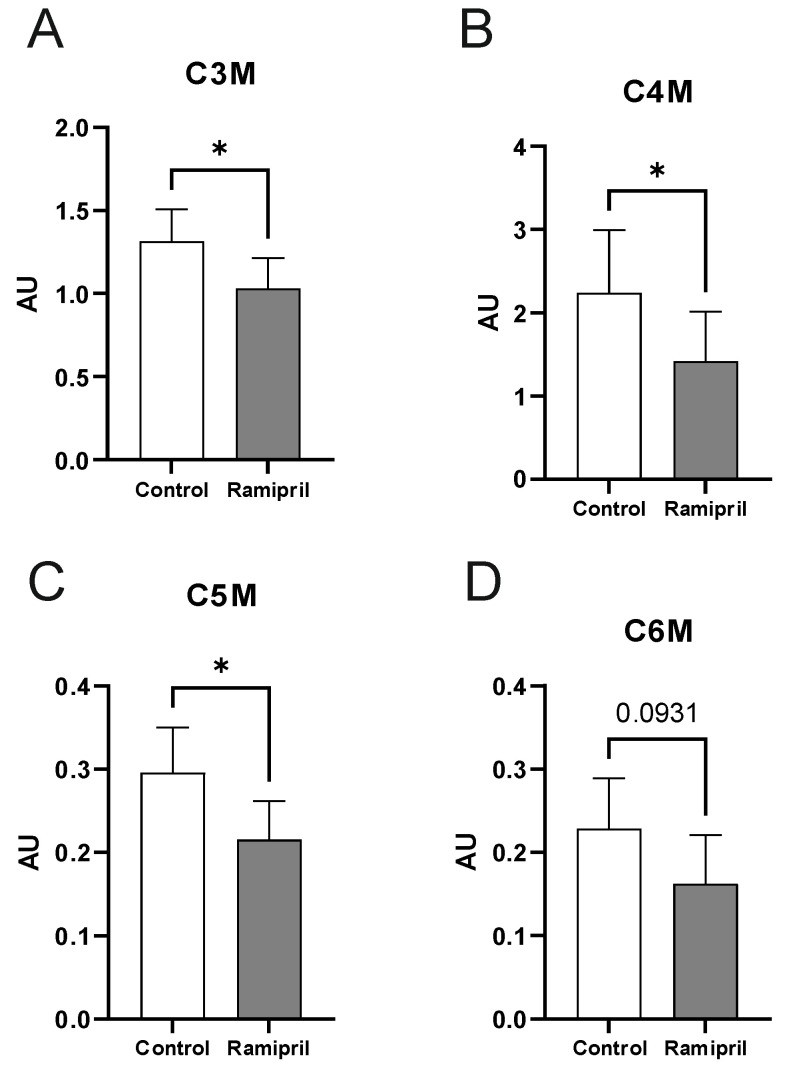
Serum metabolomics. Levels of circulating acylcarnitines: (**A**) propionylcarnitine-C3M, (**B**) Butyrylcarnitine C4M, (**C**) isovalerylcarnitine C5M, and (**D**) hexanoylcarnitine C6M (*n* = 6 per experimental group) in controls and Ramipril-treated rats. Results are expressed as means ± SD of an age of 12 weeks per experimental group. The different degrees of significance were indicated as follows: * *p* < 0.05. Differences among the groups were performed using the *Mann–Whitney test* with Graph Pad Prism; data are not normally distributed. Abbreviations: M, metabolite; AU-arbitrary units.

**Figure 3:**
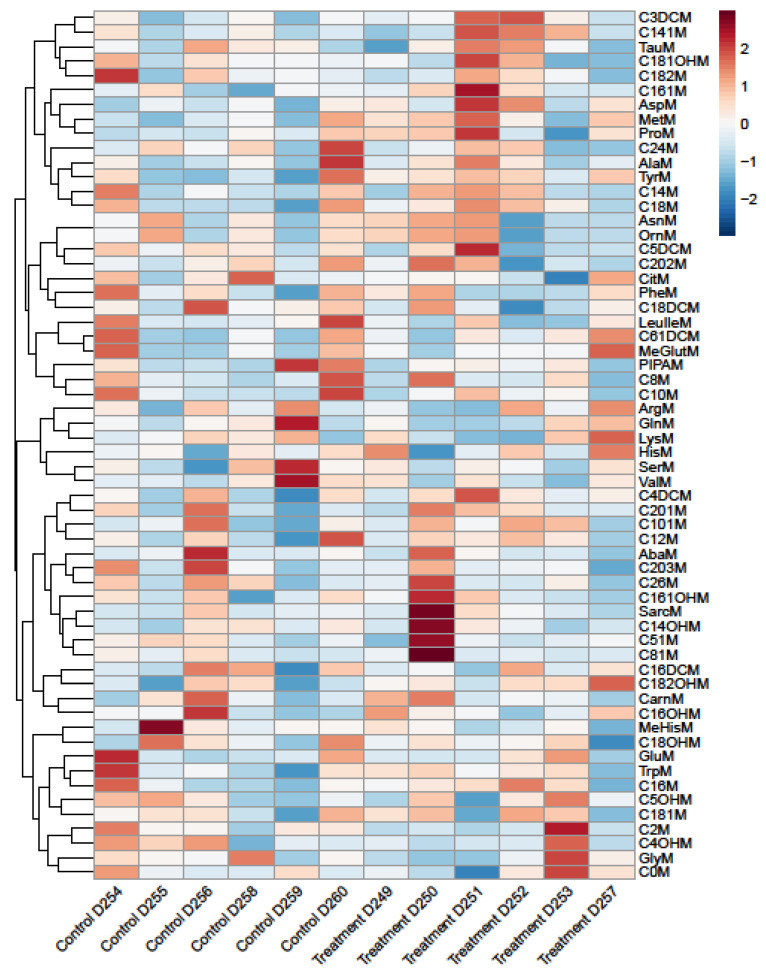
Heat Map of Metabolome (Ramipril, *n* = 6; Control, *n* = 6).

Metabolome analysis revealed a significant reduction of propionylcarnitine, butyrylcarnitine, and isovalerylcarnitine (C3M, C4M, C5M) in the Ramipril-treated rats compared to the controls (Figure 2A–D). All other metabolites were not affected by Ramipril treatment (Figure 3).

## 3. Discussion

In humans, the ACE2 receptor is abundantly expressed in lung epithelia, the small intestine, and in arterial and venous endothelial cells [9,14]. Specifically, in diabetes, ACE2 seems to play a protective role in the progression of cardiovascular and renal complications and has therefore been suggested as a therapeutic target for the management of diabetes complications [10]. Studies in diabetic mice show the role of circulating ACE2 in improved parameters of glycemia through direct effects on the pancreas [14], improving insulin sensitivity and glucose-mediated insulin release [15], and a reduced risk of developing diabetes [16]. It has therefore been hypothesized that conditions associated with ACE2 receptor overexpression, including cardio-metabolic diseases and treatment with RAAS inhibitors, may contribute to a higher COVID-19 susceptibility [17]. In addition, ACE2 receptor tissue distribution and increased expression may represent the mechanistic link underlying the reported clinical associations between the severity and outcomes of COVID-19 and hypertension, obesity, diabetes, and cardio-pulmonary diseases [18,19].

We show here that Ramipril treatment for seven days leads to increased ACE2 receptor expression in the lungs of BB/OKL rats, a model for the spontaneous development of diabetes [20,21]. It is noteworthy that a higher ACE2 receptor lung expression was not associated with higher circulating ACE2 serum concentrations after Ramipril treatment as compared to the controls (Figure 1C), suggesting that ACE2 expression in the lungs is not a major source of circulating ACE2. Together with ACE2, SARS-CoV-2 employs the androgen-regulated transmembrane protease serine 2 (TMPRSS2) as the key molecular complex to infect host cells. In this context, we find that *Tmprss2* and *Ace1* mRNA were increased in the lungs, kidneys, and duodenums of the rats treated with Ramipril as compared to the controls (Figure 1D–E). We find that *Ace1* mRNA in the heart and skeletal muscle is significantly reduced after Ramipril treatment (Figure 1E). To the best of our knowledge, such ACE inhibitor effects have not been reported previously. In contrast, we find that upon Ramipril treatment, *Ace1* mRNA expression was higher in the lungs, kidneys, and duodenum. Our observation indicates an effect of ACE inhibitor treatment on the transcription of *Ace1* that is tissue-specific. We hypothesize that upregulated *Ace1* expression in the lungs, kidneys, and duodenum may represent the effects of a positive feedback loop in response to Ace inhibition.

The focus on Ramipril treatment is a limitation of our study. Additional treatment groups for other ACEi’s and angiotensin receptor blockers (ARBs) would have been valuable to see whether our findings are specific for Ramipril or reflect the effects of other ACEi´s and ARBs. However, we obtained approval from the local Animal Welfare and Ethics Board only for the Ramipril treatment group. Yet, among the various Ace inhibitors, Ramipril is the most widely used anti-hypertensive medication in Germany [22].

We also find that intermediate metabolites of branched-chain amino acid metabolism are significantly downregulated by Ramipril treatment. In mitochondria, acyl-CoA dehydrogenases catalyze the initial step in each cycle of fatty acid *β*-oxidation, and their reduced activity leads to the accumulation of acyl-CoAs in the mitochondria [23]. These acyl-CoAs may then be scavenged into acylcarnitines that subsequently leave the mitochondrion and reach the peripheral circulation [23]. Specifically, propionylcarnitine (C3), C4-dicarboxylcarnitine (C4), and isovalerylcarnitine (C5) are produced during increased metabolism of leucine, isoleucine, and valine [24,25] and there are correlations of acylcarnitines to surrogate markers of insulin resistance [26,27,28]. Acylcarnitines also directly reflect the oxidation rate of fatty acids and amino acids. Human studies show that branched-chain amino acid-derived C3- and C5-carnitine, together with FA-derived C6- and C8-carnitine, were higher in obese and DM2 subjects compared with lean controls [29,30].

As a limitation of our experimental approach, we acknowledge that we were not able to separate isomers with our flow injection analysis; this is a compromise inherent in our high-throughput profiling method. We did not apply a chromatographic separation of amino acids to check for matrix effects on branched-chain intermediates as has been proposed in previous studies using targeted UPLC-ESI-MS/MS metabonomic analysis [31,32]. Our results may serve as pilot observations that need to be validated and further explored with more sophisticated and targeted methods in future studies.

Therefore, we conclude that the previously reported beneficial health effects of Ramipril are at least partially mediated by decreased circulating C3M, C4M, and C5M biomarkers. Importantly, Ramipril-associated changes in the serum metabolome of BB/OKL rats are specific to these acylcarnitines, and more than 60 other tested metabolites were not altered by treatment with this ACE inhibitor.

## 4. Material and Methods

### 4.1. Animals and Experimental Design

All animal studies were approved by the local authorities of the state of Saxony, Germany, as recommended by the responsible local animal ethics review board (Approval No: TVV15/20, Landesdirektion Leipzig, Germany). The Institute´s animal model platform staff looked after the animals continuously and the animals were monitored daily during regular visits from the veterinarian. Male BioBreeding/OKL rats were randomly divided into a Ramipril (10 mg/kg body weight, oral, Delix Protect, Sanofi) treatment group or a control group (*N* = 12, *n* = 6 per experimental group, oral water) over a period of 7 days. Ramipril or saline treatment was orally intragastric, administered by buttoned cannula once daily between 7 to 9 am. Phenotypes of the two experimental groups are shown in Table 1. Adiposity index (AI) and HbA1c levels were measured at the end of study. Lung, heart, skeletal muscle (quadriceps), kidney, and duodenum tissue samples were collected immediately after euthanasia by an overdose of isoflurane followed by cervical dislocation.

### 4.2. Molecular and Protein Analysis

RNA isolation and quantitative real-time PCR (qPCR) were performed using the standard curve method as previously described [33,34]. The probes, *Ace1* (Rn00561094_m1), *Ace2* (Rn01416289_m1), *Tmprss2* (transmembrane protease serine 2, Rn00590459_m1) and 18sRNA (Hs99999901_s1, endogen reference) were purchased from Life technologies (Darmstadt, Germany) and span exon-exon boundaries. Membrane proteins of the duodenum, kidneys, heart and lungs (*n* = 5/per group) were isolated using MembraneMax™ Protein Expression Kits (ThermoFisher Scientific, Germany). Proteins were detected by incubating with HRP-conjugated secondary antibodies at a 1:3000 dilution (Dianova, Hamburg, Germany) at room temperature for 2 h and a chemiluminescence kit (Amersham Pharmacia Biotech, Freiburg, Germany). Equal protein loading was verified using mouse anti-ß-actin (2 μg/mL; #3700; Cell Signaling Technology). ACE2 serum concentrations were measured by ELISA using rat standards according to the manufacturer’s protocol (Angiotensin I Converting Enzyme 2 ELISA; Cloud-Clone Corp., #SEB886Ra, Houston, TX). Circulating HbA1c percent levels were measured using the Hitado Super ID (Möhnesee, Germany). For analysis SigmaStat (Jandel Scientific, San Rafael, CA, USA) was used.

### 4.3. Metabolome Analysis

Mass spectrometric analysis of amino acids (AS) and acylcarnitines (AC) was performed using a SCIEX Triple Quad 4500 System (AB SCIEX, Darmstadt, Germany) with Turbo Ion Spray Source (TIS) in combination with a HTC Pal autosampler and a Shimadzu UFLC system for flow injection analysis (FIA) according to a validated protocol [35]. Briefly, 10 µL serum was diluted 1:10 with methanol. After centrifugation, 10 µL of the supernatant was diluted with 100 µL of methanol-containing isotope-labeled standards (Chromsystems Germany). Samples were evaporated at 70 °C for 40 min and derivatized using 60 µL of 3 n butanolic-HCL (Chromsystems, Germany) at 65 °C. After evaporation the samples were reconstituted with 150 µL of the mobile phase (1/1 *v*/*v* methanol/water) analysed with a SCIEX 4500 quadrupole tandem mass spectrometer in multiple reaction monitoring (MRM). Concentrations of 26 AAs, 34 ACs, and free carnitine were quantified using ChemoView™ 1.4.2 software (AB SCIEX, Darmstadt, Germany).

### 4.4. Statistical Analyses

The statistical data analyses were performed using GraphPad Prism 9 Software (Jandel Scientific, San Rafael, CA). Differences among the groups (*n* = 6) were performed using one-way-ANOVA, the Newman–Keuls test, and the Student’s *t*-test (normally distributed data) or the Mann–Whitney test (not normally distributed data). Results are presented as means ± SEM (Figure 1) and means ± SD (Figure 2). Statistical significance was accepted at *p* < 0.05. The different degrees of significance were indicated as follows: * *p* < 0.05, ** *p* < 0.01, *** *p* < 0.001.

## 5. Conclusions

Taken together, we provide in vivo evidence from a rat model that short-term ACE inhibitor treatment selectively increases *Ace2* receptor mRNA and membrane protein expression in the lungs and, in parallel, down-regulates the branched-chain intermediate metabolism. Whether increased ACE2 expression in the lungs occurs in humans upon long-term ACE inhibitor therapy, and whether and how that might affect COVID-19 severity, remain the major open questions that our study aimed to stimulate.

## Figures and Tables

**Table 1:** Characteristics of experimental groups before (pre) and after (post) Ramipril treatment.

	Controls (*n* = 6)		Ramipril-Treated (*n* = 6)		
	Pre	Post	Pre	Post	
Body weight (g)	360 ± 18	368 ± 17	338 ± 18	341 ± 18	n.s.
HbA1c (%)	4.2 ± 0.1	4.2 ± 0.1	4.2 ± 0.2	4.4 ± 0.3	n.s.
Adiposity Index (AI)		1.7 ±0.4		2.0 ± 0.3	n.s.

n.s.: not significant within the experimental groups before and after treatment or between controls and Ramipril-treated animals.

## Data Availability

The data presented in this study are available via e-mail from the corresponding and first author.
